# Dust at Various Workplaces—Microbiological and Toxicological Threats

**DOI:** 10.3390/ijerph15050877

**Published:** 2018-04-27

**Authors:** Beata Gutarowska, Justyna Szulc, Adriana Nowak, Anna Otlewska, Małgorzata Okrasa, Anita Jachowicz, Katarzyna Majchrzycka

**Affiliations:** 1 Institute of Fermentation Technology and Microbiology, Lodz University of Technology, 90-924 Łódź, Poland; beata.gutarowska@p.lodz.pl (B.G.); adriana.nowak@p.lodz.pl (A.N.); anna.otlewska@p.lodz.pl (A.O.); jachowicz.anita@gmail.com (A.J.); 2 Department of Personal Protective Equipment, Central Institute for Labour Protection—National Research Institute, 90-133 Łódź, Poland; maokr@ciop.lodz.pl (M.O.); kamaj@ciop.lodz.pl (K.M.)

**Keywords:** dust, microorganisms, metagenome analysis, cytotoxicity, workplaces

## Abstract

The aim of the present study was to evaluate the relation between the chemical (analysis of elements and pH) and microbiological composition (culture and metagenomics analysis) of the dust at various workplaces (cement plant, composting plant, poultry farm, and cultivated area) and the cytotoxicity effect on the human adenocarcinoma lung epithelial adherent cell line A-549 (MTT assay test). Analysis of the Particulate Matter (PM) fraction showed that the dust concentration in cultivated areas exceeded the OELs. For the remaining workplaces examined, the dust concentration was lower than OELs limits. The number of microorganisms in the dust samples was 3.8 × 10^2^–1.6 × 10^8^ CFU/g bacteria and 1.5 × 10^2^–6.5 × 10^6^ CFU/g fungi. The highest number of microorganisms was noted for dust from cultivated areas (total number of bacteria, actinomycetes, *P. fluorescens*) and composting plants (xerophilic fungi and staphylococci), while the least number of microorganisms was observed for dust from cement plants. Many types of potentially pathogenic microorganisms have been identified, including bacteria, such as *Bacillus*, *Actinomyces*, *Corynebacterium*, *Prevotella*, *Clostridium*, and *Rickettsia*, and fungi, such as *Alternaria*, *Cladosporium*, *Penicillium*, and *Aspergillus*. The most cytotoxic to the human lung cell line A-549 was dust from cultivated areas (IC_50_ = 3.8 mg/mL after 72 h). The cytotoxicity of the tested dust samples depends on the PM concentration, the number of microorganisms, including potentially pathogenic genera, and the exposure time.

## 1. Introduction

High dust concentrations affect employees in many branches of industry, primarily mining, quarrying, tunnelling, stone masonry, building construction, foundries and other metallurgical processes, glass and ceramics manufacturers; handling of powdered chemicals in various manufacturing practices; agricultural work involving exposure to soil, intensive animal husbandry, dry vegetable products, and food processing, and any process involving weighing, bagging, bag-emptying or the dry transport of powdered or friable materials [1].

Dust includes particulate matter (PM), ranging from 0.001 to 100 µm in aerodynamic diameter. which is divided into various fractions: PM_10_ (<10 µm), PM_2.5_ (<2.5 µm), and ultrafine (<0.1 µm) [2]. Dust particles, depending on the diameter and shape, can be deposited in various parts of the respiratory system. Particles with a diameter of 5 to 10 µm are mainly deposited in nasopharyngeal areas, and particles larger than 0.5 µm in diameter are deposited in bronchi, bronchioles and alveoli. Respirable dust, with a diameter less than 2.5 µm, can pass into the lungs and the bloodstream. Particles from the lower parts of the respiratory system are slowly removed (up to several hundred days) due to the lack of ciliated epithelium [3]. 

Chemically dust contains complex mineral substances, particularly metals (Cu, Cr, Fe, Zn, etc.), solid particles of plant and animal origin (allergens, including pollens, fibres, epidermis, etc.), and viable and non-viable macro- and microorganisms with the substances they release (including endotoxins, glucans, mycotoxins, peptidoglycans, enzymes, volatile organic compounds, etc.), making dust a major source of harmful biological agents [4]. The toxic effect of dust collected both inside and outside of buildings may depend on the components present in the dust, particularly its organic fraction, including microorganisms, which have great importance [3]. 

Mesophilic bacteria, actinomycetes and moulds, including pathogenic and toxinogenic species, are primarily found in dusty workplaces. Many moulds identified in composting plants, power plant processing plant biomass and agricultural area workplaces (e.g., *Alternaria*, *Aspergillus*, *Fusarium*) are recognised as allergenic strains [5]. Microorganisms from groups 2 and 3, according to Directive 2000/54/EC (e.g., *Bacillus anthracis*, *Salmonella choleraesuis* var. Typhi, *Aspergillus fumigatus*, *Candida albicans*, *Cryptococcus neoformans*, *Listeria monocytogenes*, *Mycoplasma* sp. *Staphylococcus* sp., and *Streptococcus* sp.), are often detected in the manure of industrial poultry farms [6]. In the case of cement plants, there are no data on the microbial composition of dust. 

Dust of organic origin dust, for example, coal, dust from herbs, flax scotching and animal farms, particularly poultry production and swine feed industry, biomass used for power generation, dust from biofuel plants, wood dust, dust from waste collection and sorting, and dust in sludge drying units, is a serious threat to the health of workers [7,8,9]. Additionally, inorganic origin dust, such as cement dust, can be irritative to proinflammatory and cytotoxic effects, and workers in aluminium manufacturing facilities are also exposed to fine inorganic dust [10].

Dust occurring in the air at workplaces easily penetrates the respiratory track of workers. Notably, dust could be responsible for inducing toxicity, irritation, allergies, and cancer or fibrosis, and results in diseases, such as chronic obstructive lung disease, asthma, chronic bronchitis, bronchial hyperreactivity, organic dust toxic syndrome, and irritation of the mucous membranes of the conjunctiva and skin [11]. 

Studies of the cytotoxicity and genotoxicity of urban and occupational PM in mammalian cells have shown that exposure to PM can result in increased cell death (apoptosis and necrosis), increased levels of DNA strand breaks, oxidative damage and toxicity as a result of generation of reactive oxygen species (ROS) [12]. Toxic quantitative and qualitative effects of PM on mammalian cells depend on the PM size [2], chemical and gravimetrical composition of PM [13]. 

Since many working environments have not been characterised thus far, particularly the microbiological contamination of dust present in workplaces and toxic effects on human lung cells, the aim of the present study was to evaluate the relationship between chemical and microbiological composition of dust at various workplaces and the cytotoxicity effect on the human adenocarcinoma lung (alveolar) epithelial adherent cell line A-549. For this purpose, the PM concentration at the workplaces, including the two cement plants, composting plant, poultry farm, and cultivated area, was analysed, and the elements, pH, microbial contamination (by using a culture method for culturable microorganisms and a molecular method, i.e., metagenomics analysis, for unculturable microorganisms) as well as the cytotoxicity of dusts collected from tested workplaces were investigated.

## 2. Materials and Methods

### 2.1. Working Environments

Analysis was performed at four working environments: (1) cement plants (two sampling places), (2) a composting plant, (3) a poultry farm and (4) a cultivated area located in Poland. Working environments were selected based on the data of high organic/inorganic dust [1]. Table 1 and Table 2 summarise the characteristic of the tested working environments. Temperature, relative humidity and airflow rate were measured by using a thermo-anemometer VelociCalc^®^ Multi-Function Velocity Meter 9545 (TSI, Shoreview, MN, USA). 

### 2.2. Airborne Dust Concentration Measurement

The airborne dust concentration at tested workplaces was measured by using a DustTrak™ DRX Aerosol Monitor 8533 portable laser photometer (TSI), which enabled simultaneous measurements of size-segregated mass concentrations corresponding to PM_1_, PM_2.5_, PM_4_, PM_10_ and total PM size fractions. The detection range of the instrument was from 0.001 to 150 mg/m^3^ for particles from 0.1 to 15 μm in size. Prior to each experiment, zero calibration was performed. The measurements were obtained at a height of 1.5 m from ground level in triplicate for each location, with a sampling rate of 3 L/min and a sampling interval of 1 s.

### 2.3. Settled Dust Sampling

Settled dust samples (part of airborne particulate matter that fell down onto sampling surface during work activities) were collected onto five glass plates. The sampling plates were set on the height of about 1.5 m and left at the workplace for 24 h. The amount of settled dust ranged between 10 and 20 g. Next, samples were carefully swept with disposable brush into polypropylene string bags, then mixed together and used for further analyzes.

### 2.4. Chemical Composition of Settled Dust

The elemental composition of the settled dust collected at tested workplaces, considering carbon, nitrogen, hydrogen, sulfur and phosphorus contents, as well as the C:N ratio, determined by using an elementary analyser (type NE 2500, CE Instruments, Wigan, UK). Moreover, the pH of the dust suspension (0.5 g of each dust sample was suspended in 5 mL of basal medium for culturing the A-549 cells, under conditions described in cell culture and cytotoxicity testing) was established by using a CP-401 pH meter (Elmetron, Zabrze, Poland).

### 2.5. Microbial Contamination 

#### 2.5.1. Culture Methods

Samples of settled dust from working environments were microbiologically analysed. For this purpose, 10–40 g samples of settled dust were mixed, and a 0.1 g sample was suspended in 9.9 mL of saline solution (0.85% NaCl). The samples were diluted from 10^−2^ to 10^−6^ in duplicates and plated onto MEA (Malt Extract Agar, Merck, Darmstadt, Germany) medium with (0.1%) chloramphenicol (fungi); DG18 Agar (DG18 LAB-AGAR™, Biocorp, Warszawa, Poland) (xerophilic fungi); TSA (Triptic Soy Agar, Merck) with (0.2%) nystatin (bacteria); Columbia Blood Agar, (Oxoid, Dardilly Cedex, France) (haemolytic staphylococcus); Pochon’s agar (Labomix, Łódź, Poland) with (0.2%) nystatin (actinomycetes); Chapman Agar (Merck) (mannitol-positive *Staphylococcus* spp.); King B medium (Hi Media Laboratories, Mumbai, India) (*Pseudomonas fluorescens*); and Violet Red Bile Glucose Agar (VRBG LAB-AGAR, Biocorp) (*Enterobacteriaceae*). The samples were incubated at 37 ± 2 °C for 24–48 h (*Enterobacteriaceae*, mannitol-positive *Staphylococcus* spp.), at 25 ± 2 °C for 5–7 days (fungi, xerophilic fungi, actinomycetes), or at 30 ± 2 °C for 48 h (bacteria, *Pseudomonas fluorescens*). After incubation, the colonies were counted, and the results were expressed in CFU/g (CFU—colony forming unit) of dust. The data were analysed from three independent experiments. The final result was calculated as the arithmetic mean and standard deviation (SD) of all repetitions.

#### 2.5.2. Metagenomics Survey of Microbial Populations

DNA was extracted from 0.5 g of each settled dust samples by using the FastDNA^®^ Spin Kit (No. 116560200, MP Biomedicals, Santa Ana, CA, USA) according to the manufacturer’s protocol. The quantification of DNA was conducted by using a Qubit 2.0 Fluorometer (Invitrogen/Life Technologies, Carlsbad, CA, USA). The bacterial 16S rRNA gene was amplified by using universal prokaryotic primers, 341 F and 785 R targeting the V3–V4 regions. The fungal ITS1 region was amplified with ITS1F12 forward [14] and ITS2 reverse primers [15]. All PCR reactions were performed in triplicate in 50 μL, containing 5 µL of DNA as a template, 25 µL of NEBNext^®^ Hot Start High-Fidelity 2 × PCR Master Mix (New England BioLabs, Ipswich, MA, USA) and 10 pmol of each primer. The amplification of bacterial and fungal fragments was performed under the same conditions, with an initial denaturation at 98 °C for 30 s, followed by 15 cycles of denaturation at 98 °C for 10 s, annealing at 52 °C for 75 s, and extension at 65 °C for 75 s, with a final extension at 65 °C for 5 min. DNA libraries were constructed by using the Nextera Index Kit according to the library preparation protocol for short amplicons (2 × 250 bp) provided by the manufacturer. Paired-end (PE, 2 × 250 nt) sequencing was performed on the Illumina MiSeq (MiSeq Reagent kit v2) according to the manufacturer’s protocol (Illumina, Inc., San Diego, CA, USA) at Genomed (Warsaw, Poland).

QIIME was used to determine the microbial communities in different types of dust [16]. The raw reads were demultiplexed and quality-filtered on MiSeq by using MiSeq Reporter (MSR) v2.4 (BaseSpace, San Diego, CA 92122, USA). The sequences were clustered based on 97% identity by using the uclust algorithm [17], and OTUs (Operational Taxonomic Units) were assigned to taxa employing the GreenGenes database v13_8 for bacteria and UNITE v7 database for fungi [18].

### 2.6. Cell Culture and Cytotoxicity Testing

Next, each dust sample (0.5 g) was suspended in basal medium (5 mL) for culturing the cells (described later) and mixed well (the stock dust concentration in each extract was 100 mg/mL). The samples were then extracted for 40 min (160 rpm) at an ambient temperature. Alkaline or acidic pH of the test substance could induce the detachment of the cells from the monolayer and cytotoxicity; to exclude these effects, the pH of each extract was conveyed to neutral (pH 7.0 ± 0.2), and each extract was twice filtered by sterile syringe filters (0.22 μm, Membrane Solutions, Kent, WA, USA). The final concentrations of the extracts analysed were 20, 10, 5, 2.5, 1.3, 0.6 and 0.3 mg/mL.

The cytotoxicity of the prepared water-soluble fraction of dust samples was assessed by an MTT (3-(4,5-dimethylthiazol-2-yl)-2,5-diphenyltetrazolium bromide) assay, while for extracts 5 and 6, cytotoxicity was determined with a NRU (neutral red) assay. The assays were performed on the human adenocarcinoma lung (alveolar) epithelial adherent cell line A-549 (Cell Line Service GmbH, Eppelheim, Germany) from passage 33. The model cell line stimulates conditions close to the actual finest ingress fractions of particulate matter pollutions in alveoli and is often used for dust and air pollution cytotoxicity testing [2]. 

The cells were cultured in flasks of Roux type (Greiner Bio-One GmbH, Frickenhausen, Germany) as a monolayer in Dulbecco’s Modified Eagle’s Medium:Ham’s F12 basic (1:1, *v/v*, DMEM/Ham’s F12, Cell Line Service GmbH) complemented with 5% foetal bovine serum (FBS, Cell Line Service GmbH), 2 mM of glutamine (Cell Line Service GmbH), 25 mM of HEPES (Cell Line Service GmbH), 100 μg/mL streptomycin and 100 IU/mL penicillin (Sigma-Aldrich, St. Louis, MO, USA). The cells were cultured for 3–5 days at 37 °C in an incubator with a 5% CO_2_ atmosphere (Galaxy 48S, New Brunswick, UK). The cells were washed every 2 days with phosphate-buffered saline (PBS, Sigma-Aldrich, St. Louis, MO, USA, pH 7.2), and the medium was exchanged for fresh medium. After reaching 80% confluence, the cells were detached with TrypLE^TM^ Express (Gibco, Thermo Fisher Scientific, Waltham, MA, USA) for 3–5 min at 37 °C and gently shaken from the plastic flask. The cell suspension was then centrifuged (187× *g*, 5 min), the pellet was re-suspended in fresh culture medium, and subsequently, and the cell count was performed using a haemocytometer. The viability of the cells was determined by trypan blue exclusion. The cells were ready to use if they showed a minimum of 80–90% viability.

For both MTT assays, 1 × 10^4^ A-549 cells were placed in each well of 96-well plate (Greiner Bio-One GmbH) in the complete culture medium. The cells were incubated overnight at 37 °C in 5% CO_2_. The following day, the medium was gently aspirated, and 200 µL of each concentration of the tested extract in the culture medium was added to each well in four repeats. The negative controls (in eight repeats) consisted of cells without tested extracts. The cells were incubated in a CO_2_ incubator at 37 °C in 5% CO_2_ for 48 and 72 h. After incubation, the medium with extracts was gently aspirated, and 100 µL of MTT (0.5 mg/mL in PBS, pH 7.2) (Sigma-Aldrich) was added and incubated at 37 °C in 5% CO_2_ for an additional 3 h. MTT was then carefully removed and formazan precipitates were solubilised by adding 50 µL of DMSO (Sigma-Aldrich). Absorbance was measured at 550 nm with a reference filter of 620 nm, by using a microplate reader (TriStar2 LB 942, Berthold Technologies GmbH and Co. KG, Bad Wildbad, Germany). The absorbance of the control sample (untreated cells) represented 100% cell viability. Cell viability (%) was calculated using the following equation: (sample OD/control OD) × 100%; and cytotoxicity (%) as: 100—cell viability (%). The results are presented as the means ± standard deviation (SD). The mean error of these methods is up to 10%.

The IC_50_ values (the concentration of the test compound required to reduce the cell survival rate to 50% of the control) were considered as a degree of cellular sensitivity to a given treatment. IC_50_ values were read from the achieved curves and additionally determined according to the OECD Guidelines for the Testing of Chemicals [19,20].

### 2.7. Statistical Analysis

Selected microorganisms in settled dust as well as for the granulometric fractions of dust from different sampling places were described using means, standard deviation and range. Statistical analyses were conducted by using STATISTICA 13.3 software (Statsoft, Kraków, Poland). The Shapiro-Wilk test was performed to assess the normality of the distribution of each variables of interest. When distribution was normal the results were evaluated by using one-way analysis of variance (ANOVA) at a significance level of 0.05. When the significant difference was detected (*p* < 0.05), the means were compared by using Tukey’s post hoc procedure at a significance level of 0.05. Otherwise, a non-parametric Kruskal-Wallis test at a significance level of 0.05 was performed followed by Dunn’s post hoc multiple comparisons (*p* < 0.05).

## 3. Results and Discussion

### 3.1. Airborne Dust Concentration at Workplaces

Airborne dust fraction PM_1_, PM_2.5_, PM_4_ and PM_10_ concentrations did not significantly differ for workplaces No. 1–5 (*p* > 0.05, Table 3). 

The total airborne dust concentration was higher at workplace No. 5, where the dynamic movement of the organic dust was observed during grain transportation to the silo by using a blower. There were no significant differences between workplaces No. 1–3, where the lowest concentration values were noted due to low to no dynamics of the processes that occurred therein.

At workplaces No. 1 and 3, the dominant PM fraction had an aerodynamic diameter below 1 µm, accounting for 87.3% and 80.8% of the total dust concentration. At workplaces No. 2, 4, and 5, dust particles with aerodynamic diameter below 1 µm, respectively accounting for 30.8%, 27.4% and 59.7%, and PM with aerodynamic diameters larger than 4 µm constituted respectively 63.7%, 68.6% and 38.4% of the total measured dust concentration. In all cases, the smallest portion of the total PM constituted particles with diameters between 1 and 4 µm (1.8–6.2%). 

Differences in PM deposition sites and retention times are among the main factors determining the toxicity of inhaled particles. Thus, the principle of particle size-selective thresholds in their occupational exposure limits (OELs) is commonly accepted. In Europe, there are two types of OELs: EU community exposure limits set by the European Agency for Safety and Health at Work and national exposure limits individually established by the Member States. Moreover, the EU and national exposure limit values may be different from those established in non-European countries. For example, the 8-h threshold limit of inhalable dust (PM) concentration varies from 4 mg/m^3^ (Germany, insoluble particulates) to 15 mg/m^3^ (USA-OSHA). For respirable dust, the 8-h OELs are between 1.25 mg/m^3^ (Germany, insoluble particulates) and 6 mg/m^3^ (Hungary). OELs may also significantly differ depending on the specific type of dusts. For organic dusts such as grain dust, 8-h OELs vary from 1 mg/m^3^ (Japan-JSOH) to 10 mg/m^3^ (Ireland, USA-OSHA and United Kingdom) [21].

Cement dust exposure of cement plant workers has previously been documented. However, the results widely vary, depending on the cement production technology, climatic conditions and type of activities performed by the workers. Other studies have reported that the average 8-h concentration of respirable cement dust can range from 0.4 to 38.6 mg/m^3^ throughout the production process [22,23]. The present results are consistent with the lower values reported by previous studies. The measurements were obtained in the conveyor hall during the transportation of the clinker; thus, the movement of the fired mass and surrounding air was rather limited, which contributed to the low values of the measured PM concentration.

The exposure of workers to dust in composting facilities has also been assessed due to occupational risks posed by biologically reach organic PM and its increased toxic potential. Viegas et al. performed measurements of size-segregated mass concentrations corresponding to PM_0.5_, PM_1_, PM_2.5_, PM_5_, and PM_10_ size fractions at two Portuguese compositing plants [24]. PM data showed higher contamination for PM_5_ and PM_10_ fractions (approx. 0.1–1.0 mg/m^3^) and lower contamination for smaller fractions (below 0.1 mg/m^3^). Pearson et al. reviewed 13 different occupational studies measuring personal exposure of compost plant workers to different bioaerosol components and determined that organic dust levels may vary from 0.1 to even 51.14 mg/m^3^ depending on the compost site operations [25]. The results reported in the present study show the same tendency in particle size distribution and PM concentration to those previously reported. Low values of PM concentration may be associated with low temperature and high relative humidity that limit the convective movements of airborne particles and the high humidity of the composted waste materials reducing dust generation.

Ellen et al. and Viegas et al. performed studies to determine dust concentrations in poultry farms. The authors found similar results, where PM_10_ predominated at concentrations between 1.4 and 15.2 mg/m^3^ [26,27]. Skóra et al. conducted another study at a poultry farm, reporting similar but slightly lower values for the concentration between 0.48 mg/m^3^ for PM_1_ and 0.875 mg/m^3^ for PM_10_ [28]. Comparable particle size distribution and concentrations were observed in the present study, where larger particles contribute to the overall mass concentration. Slight differences in concentrations of airborne dust observed at different locations may result from the stage of the production cycle, type of birds, indoor ventilation system and a multitude of external factors, such as ambient temperature and relative humidity.

Recent literature data on the PM emissions resulting from the grain harvesting is rather sparse and typically concerns ambient air measurements over areas surrounding the fields with harvested crops [29]. Notably, the highest concentration of PM_total_ within the present study were taken during the activities with the most rapid movement of the grain material through the air, thus causing the increased generation of dust. To fully understand the exposure levels expected along the entire harvesting process, comprehensive studies considering the array of activities performed by the workers are necessary. Despite the need for more detailed data, the information obtained within the present study highlight the importance of preventive and protective measures that should be undertaken in such work processes.

### 3.2. Chemical Composition of Settled Dust

Chemical composition of the sampled dust varied widely across workplaces. The most variation was observed in carbon content and the least in hydrogen content: carbon 1.5% (dust No. 2, cement plant) to 71.2% (dust No. 4 from poultry farm); hydrogen, 1.4% (dust No. 2, cement plant) to 5.9% (dust No. 4), (Table 4). 

Only traces of sulfur and phosphorus were found in the tested dusts (from 0.01 to 0.18%) except for dust No. 4, in which the sulfur content was 0.93% and phosphorus was 2.54% (Table 4). The ratio of carbon to nitrogen (C:N) in the samples tested widely varied depending on the type of dust and ranged from 3.82 to 98.64. A low ratio of carbon to nitrogen (C:N ≈ 10) was found for dust No. 1 and 2 (cement plant). A higher ratio of carbon to nitrogen was found for dust No. 4 (C:N = 14.53) and grain dust No. 5 (C:N = 29.09). The highest ratio of carbon to nitrogen (C:N = 98.64) was found for dust from composting plant No. 3. Most of the dust samples were slightly acidic. Only dust No. 2, from the cement plant, was alkaline (Table 4). 

The results obtained in the present study suggest that settled dusts No. 3, 4 and 5 contain appropriate environments for the development of microorganisms (high carbon content and adequate ratio C:N). Herron et al. tested the chemical composition of the dust emitted from poultry broiler houses. These authors noted that the pH value of dust ranged from 7.0 to 7.8, and the concentration of carbon in the studied dust ranged from 28.6 to 35.3%, while that for nitrogen ranged from 6.9 to 14.6%, and the concentration of phosphorus ranged from 1.2 to 2.1%, which corresponded to the results of the present study [30]. The isolation of respirable fraction of particles from residential house-dust made by Gustafsson et al. showed an elemental composition of 78% carbon and 2.4% nitrogen in the dust calcium, potassium, silica, sulfate, aluminium and chlorine (content: 0.3–2.0%) [3]. The content of elements in settled dust tested in the present study is comparable to results by other authors.

### 3.3. Microbial Contamination of Settled Dust

In the tested settled dust samples, bacteria were dominant, with numbers ranging from 3.75 × 10^2^ CFU/g (sample No. 2, cement plant) to 1.57 × 10^8^ CFU/g (No. 5, cultivated area). The number of fungi ranged from 1.5 × 10^2^ CFU/g (No. 2) to 6.48 × 10^6^ CFU/g (No. 3, composting plant) (Table 5). 

Among bacteria, mannitol-positive and mannitol-negative *Staphylococci* spp. predominated. The numbers of these bacteria ranged from 1.0 × 10^2^ CFU/g (No. 2) to 1.07 × 10^7^ CFU/g (No. 3), and haemolytic *Staphylococcus*: 1.0 × 10^3^ CFU/g (No. 2)–4.85 × 10^6^ CFU/g (No. 3). A lower number of bacteria *Pseudomonas fluorescens* was detected in the studies samples (2.75 × 10^3^–7.30 × 10^5^ CFU/g), except for sample No. 2, where *P. fluorescens* did not exceed the 1.0 × 10^2^ CFU/g level. In addition, *Enterobacteriaceae* did not exceed the 1.0 × 10^2^ CFU/g level in samples No. 2 and 4, whereas in other samples, the number of these bacteria ranged from 1.07 × 10^4^ CFU/g (No. 3) to 3.81 × 10^4^ CFU/g (No. 1) (Table 5). The presence of actinomycetes above the 1.0 × 10^2^ CFU/g level was only found in sample No. 5 (cultivated area). Among the fungi, many xerophilic genera were detected, from 1.50 × 10^2^ CFU/g (No. 2) to 3.45 × 10^6^ CFU/g (No. 3) (Table 5). The lowest microbiological contamination was noted for settled dust sample No. 2 (cement plant). Moreover, statistical analysis of the obtained results showed statistically higher (*p* < 0.05) total bacteria, actinomycetes and *P. fluorescens* numbers in sample No. 5 (cultivated area) compared to other tested dust samples. However, in dust No. 3 (composting plant), a statistically significant higher (*p* < 0.05) number of xerophilic fungi, mannitol-positive and negative staphylococci and haemolytic staphylococci were detected. Moreover, a statistically significant higher (*p* < 0.05) number of *Enterobacteriaceae* were found in the case of dust No. 1 (cement plant, alternative fuel hall) (Table 5).

**Figure 1:**
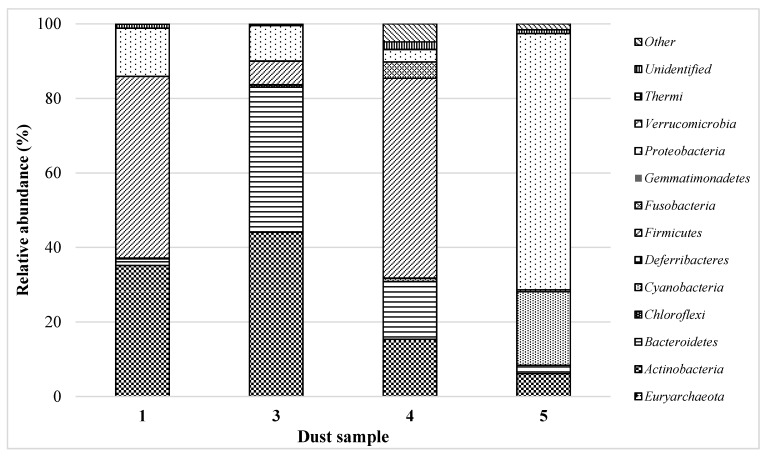
Phylogenetic distribution of bacterial sequences assigned on phyla in dust samples (**No. 1**) cement dust collected in the alternative fuel hall; (**No. 3**) dust from a composting plant collected in the homogenisation hall of waste; (**No. 4**) poultry dust collected from a livestock room—laying hens; (**No. 5**) dust from grain collected from the blower elements transporting freshly harvested wheat from the field to the silo.

**Figure 2:**
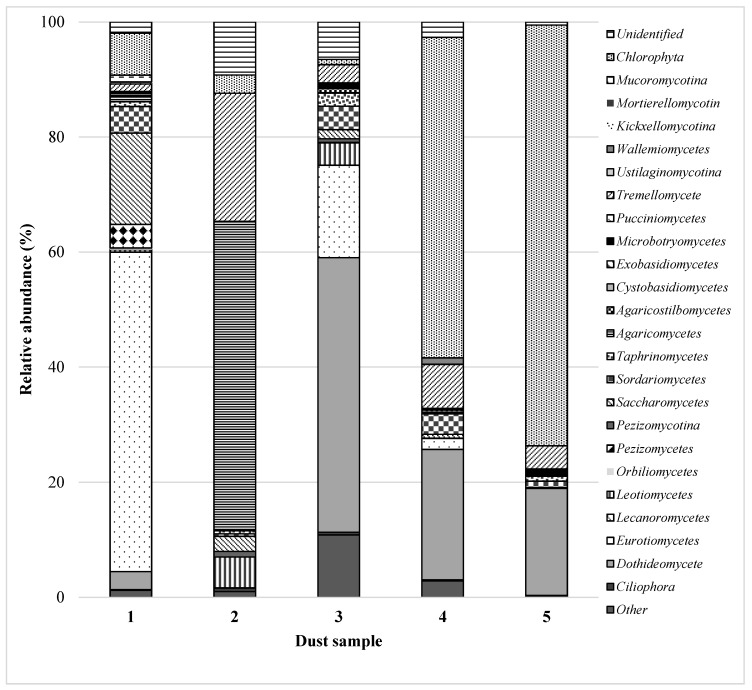
Phylogenetic distribution of fungal sequences assigned to phyla in dust samples. (**No. 1**) cement dust collected in the alternative fuel hall; (**No. 2**) cement dust collected in the clinker transporting conveyor hall; (**No. 3**) dust from a composting plant collected in the homogenisation hall of waste; (**No. 4**) poultry dust taken in a livestock room—laying hens; (**No. 5**) dust from grain collected from the blower elements transporting freshly harvested wheat from the field to the silo.

DNA high-throughput sequencing data revealed the presence of 182 bacterial genera belonging to 12 classes and 150 fungal genera from 22 classes in the cement dust (No. 1) collected from the alternative fuel hall (Figure 1 and Figure 2). This sample was mainly contaminated by bacteria from classes: *Firmicutes* (48.8%)*, Actinobacteria* (35.2%) and *Proteobacteria* (12.9%). The other classes represented a small fraction (below 2.0%) of the bacterial communities (Figure 1). 

Amongst *Firmicutes*, DNA sequences associated with bacteria of the genera *Bacillus* (16.6%), *Aerococcus* (15.0%), *Lactobacillus* (4.0%) and *Staphylococcus* (3.0%) were detected. *Actinobacteria* was mostly represented by *Corynebacterium* (13.0%), *Actinomyces* (7.4%), *Saccharomonospora* (3.7%) and *Brachybacterium* (3.4%), while *Acinetobacter* (3.7%), *Pseudomonas* (1.7%) and *Erwinia* (1.3%) were the most abundant genera belonging to *Proteobacteria* (Table S1)*.* Cement dust sample (No. 1) was contaminated by fungi belonging to *Eurotiomycetes* (55.5% of fungal OTUs) (Figure 2). Among them, *Aspergillus* (34.9%), *Thermomyces* (9.2%), *Penicillium* (6.6%) and *Talaromyces* (4.0%) were detected. 

Additionally, members of *Candida* sp. (7.4%) dominated *Saccharomycetes*, the second largest division, representing 15.9% of the total fungal OTUs (Table S2). Although, the sample No. 2 was also a cement dust, the results indicated that the fungal community structures were significantly diverse among the samples collected from different places of the cement plant. In this specimen, the fungal OTUs were assigned to 13 phyla and only 30 genera. *Agaricomycetes* (53.8%) with the genus *Boletus* (49.0%) were the major phylogenetic group. Moreover, this sample also yielded a high proportion (22.0%) of sequences corresponding to *Cryptococcus*, which belong to *Tremellomycetes*, representing 22.3% of the total OTUs pool (Table S2). In the case of cement dust No. 2, bacterial DNA amplification was unsuccessful.

Among bacterial genera identified in cement plant, *Bacillus*, *Aerococcus*, *Corynebacterium* and *Actinomyces* occurred with the highest OTU. The high temperature and the alkaline pH during cement production are limiting factors for the growth of microorganisms. The likelihood of growth in high pH environments and spore production by *Bacillus* bacteria could explain why the metagenomic analysis in the present study showed their dominance in the cement plant dust samples. However, *Bacillus* sp. is indicated as bacteria that play a key role in cement biodegradation [31]. Notably, *Actinomyces* (the most recognised: *A. israelii*, *A. viscosus*, *A. naeslundii*, *A. turicensis*, and *A. radingae*) are locally pathogenic in the oral cavity or through direct extension into fascial planes as abscesses. Systemic disease can also result through the exposure of *Actinomyces* to pulmonary tissue [32], which is particularly dangerous in the presence of organic dust. Moreover, among the genus *Corynebacterium*, the pathogenicity has been associated with many strains, such as *C. diphtheriae*, *C. pseudodiphtheriticum*, *C. ulcerans*, *C. pseudotuberculosis*, *C. xerosis* and *C. striatum* [33]. The moulds *Aspergillus* and *Penicillium* were predominant in cement dust samples and previously detected in cement environment [34]. Among fungi in the second sample from cement plant (No. 2), *Boletus* (*Agaricomycetes*) was dominant. The presence of mushroom-producing fungi in cement dust is likely related to the location of the cement plant (in a wooded area) and the sample collection time, July (intense sporulation).

In the settled dust from a composting plant (No. 3), 12 bacterial classes, containing 148 genera, were detected. Among these species, *Actinobacteria* (44.0%), *Bacteroidetes* (39.0%), *Proteobacteria* (9.5%) and *Firmicutes* (6.2%) were the most abundant phyla (Figure 1). The detected phyla are typical for the compost bacterial community and have been described in the literature. Partanen et al. identified the same phyla and additionally *Deinococcus-Thermus* was detected in compost samples by using full-length 16S rRNA gene sequences [35]. The majority of *Actinobacteria* sequences were affiliated with *Arthrobacter* (9.3%), *Kocuria* (9.2%) and *Curtobacterium* (8.0%). Amongst *Bacteroidetes* the most abundant genera were *Pedobacter* (21.3%) and *Olivibacter* (6.8%), while amongst *Proteobacteria*, it was *Devosia* (2.0%) and *Kaistobacter* (1.0%) (Table S1). A high fungal diversity was associated with the dust from the composting plants (24 classes, 145 genera) (Figure 2). The sample was dominated by moulds of the genera *Ramularia* and *Cladosporium* (class *Dothideomycetes*) and *Aspergillus* (class *Eurotiomycetes*) (Table S2). These moulds are commonly occurring genera involved in the decomposition of organic matter and are often plant pathogens [36]. The fingerprint technique and DNA libraries have also been used to identify the fungal species participating in composting [37,38]. These studies showed a phylogenetically wide spectrum of different phylotypes from the phyla *Ascomycota*, *Basidiomycota* and *Zygomycota*. Notably, *Aspergillus fumigatus* has been isolated from many samples of compost and air in compost plants. This species is harmful to human health due to its allergic, toxic, and invasive effects [39].

In the settled dust from the poultry farm (No. 4), the presence of 139 bacterial genera belonging to 8 classes and 107 fungal genera from 21 classes was revealed (Tables S1 and S2). Sequences of *Firmicutes*, *Bacteroidetes* and *Actinobacteria* were dominant in this sample and constituted 53.6%, 15.5% and 15.3% of OTUs, respectively (Figure 1). The predominant genera within *Firmicutes* were *Lactobacillus* (10.9%), *Clostridium* (8.6%), *Faecalibacterium* (6.3%), *Ruminococcus* (5.6%), *Anaerofilum* (4.8%) and *Megamonas* (4.6%). In the class *Bacteroidetes*, DNA sequences affiliated with *Prevotella* (8.5%) and *Bacteroides* (6.0%) were detected, while the genera *Adlercreutzia* (6.6%) and *Bifidobacterium* (4.6%) represented the *Actinobacteria* phylum. However, within the bacterial community, *Fusobacteria*, *Proteobacteria*, *Cyanobacteria*, *Deferribacteres*, *Thermi* and *Verrucomicrobia* were in the minority (Table S1). Most of identified bacteria from the genera *Clostridium*, *Bifidobacterium*, *Lactobacillus*, *Ruminococcus*, *Bacteroides*, *Megamonas* and *Prevotella* were described as characteristic for poultry farm environments and have been isolated by using both culture and molecular methods [40]. Bacteria *Prevotella* can be involved in various human infections, including infections of the head, neck, lower respiratory tract, central nervous system, and abdominal and female genital tract, and bacteremia [41]. A few *Clostridium* species are pathogenic for humans, particularly *C. perfringens*, *C. difficile*, *C. tetani* and *C. botulinum*, which produce a variety of toxins (mainly neurotoxins) [42].

A total of 22.7% fungal OTUs found in the dust from poultry farms belonged to the *Dothideomycetes* phylum, comprising the genera *Cladosporium* (18.7%), *Alternaria* (1.5%) and *Mycosphaerella* (1.3%). The genera *Trichosporon* (4.9%) represented *Tremellomycetes,* comprising 7.6% of the total fungal OTUs (Table S2).

Moulds *Cladosporium*, *Alternaria* and *Trichosporon* were previously isolated from the air and surfaces in poultry farms [5,11,28]. Moreover, *Trichosporon* has been recognised as an opportunistic agent of invasive infections, which colonises and proliferates in different parts of the human body, including the gastrointestinal system, respiratory tract, skin, and vagina, particularly in people with low immunity [43].

The settled dust from the grain No. 5 demonstrated the lower biodiversity of bacterial communities compared to that of the other dust samples (Figure 1). The bacterial OTUs were assigned to 83 genera belonging to only four phyla (Table S1). *Proteobacteria* and *Cyanobacteria* were the major phylogenetic groups. DNA recovered from the grain dust mostly represented *Proteobacteria* (68.8%) with *Rickettsia* (40.5%) and *Erwinia* (17.0%) dominance. Notably, the genus *Rickettsia* comprises aetiological agents of human diseases, including typhus, spotted fever, and scrub typhus [44]. The presence of *Rickettsia* in tested sample No. 5 constitutes a real health threat for people involved in work with freshly harvested wheat from dust. *Erwinia* are bacteria containing mostly plant pathogens, and its presence is typical for agriculture areas. However, O’Hara et al. described the first human isolate of *E. persicinus* from the urine of an 88-year-old woman who presented with a urinary tract infection [45]. Amongst *Cyanobacteria* (19.7%), only one genus was detected, namely, *Phormidium*. *Phormidium* (blue-green algae) has been studied with respect to toxin (saxitoxins and microcystins) production, potentially resulting in human and environmental health effects [46]. The remaining sequences corresponded to *Actinobacteria* (6.1%), *Bacteroidetes* (2.3%) and *Firmicutes* (0.5%). Among the fungal community (17 classes, 62 genera), *Dothideomycetes* (18.6%) and *Tremellomycetes* (3.9%) were in the majority (Figure 2). A similar phenomenon was observed in the dust from the poultry farm. *Alternaria* (7.3%) was the most abundant genus within *Dothideomycetes*, followed by *Mycosphaerella* (5.5%) and *Epicoccum* (2.9%). Additionally, similar to the cement dust No. 2 sequences, representing the genus *Cryptococcus*, a high proportion of the *Tremellomycetes* class was detected (Table S2). All mentioned moulds are plant pathogens; however, *Mycosphaerella* can cause Septoria tritici blotch (STB) disease in wheat and is a major economic constraint on wheat productivity [47].

In all tested settled dust samples, potentially allergenic moulds were present, including *Aspergillus* and *Penicillium* (cement and composting plants), *Cladosporium* (cement plants and poultry farms), *Alternaria* (poultry farms and cultivated areas), *Epicoccum* (cultivated areas) based on the World Health Organization and the Allergen Nomenclature Sub-Committee of the International Union of Immunological Societies data (International Union of Immunological Societies Allergen Nomenclature Subcommittee, 2018) [48].

In all settled dust samples, DNA sequences showed homology to the green algae *Chlorophyta* and constituted between 0.9% (dust No. 3) and 73.1% (dust No. 5). Sequences that could not be classified into any phylogenetic group were assigned as unclassified.

### 3.4. Cytotoxicity of Settled Dust

**Figure 3:**
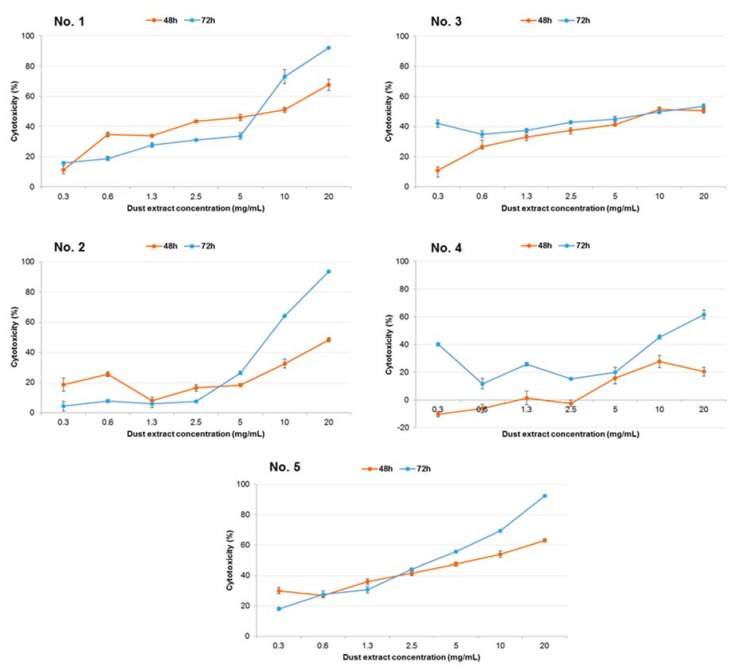
Curves representing cytotoxic activity of water-soluble fractions of dust samples in human lung A-549 cell line, evaluated in MTT assay. Each point represents the mean absorbance values from four repeats (±SD). (**No. 1**) cement dust collected in the alternative fuel hall; (**No. 2**) cement dust collected in the clinker transporting conveyor hall; (**No. 3**) dust from a composting plant collected in the homogenisation hall of waste; (**No. 4**) poultry dust taken in a livestock room, laying hens; (**No. 5**) dust from grain collected from the blower elements transporting freshly harvested wheat from the field to the silo.

The human epithelial lung cell line A-549 was challenged with the water-soluble fractions of dust samples of different origins for 48 and 72 h, with concentrations ranging from 0.3 to 20 mg/mL, in four repeats for each concentration. The curves representing the cytotoxicity of the dust samples are presented in Figure 3. 

The strongest cytotoxic dust sample was obtained from grain collected from the blower elements transporting freshly harvested wheat from the field to the silo (No. 5, Figure 3). Its cytotoxicity increased with increasing concentration, and after 72 h exposure, the cytotoxicity levels reached 92.4 ± 0.5% (for 20 mg/mL). In the presence of cement dust collected in the clinker conveyor’s hall (No. 2, Figure 3) and a poultry farm, a livestock room with laying hens (No. 4, Figure 3), the cytotoxicity was slight and adjusted up to 2.5 mg/mL. The cytotoxicity of these dust samples definitely increased from a 2.5 mg/mL concentration and accomplished values respectively for cement dust and dust from poultry farm: 93.8 ± 0.7% and 61.7 ± 3.3% after 72-h exposure (for the concentration 20 mg/mL). The cytotoxicity of dust from a hall of alternative fuels (No. 1, Figure 3) increased with increasing concentration, and after 72 h the cytotoxicity was 92.2 ± 0.3% after exposure to 20 mg/mL of the sample. Dust from the composting plant (No. 3, Figure 3) induced comparable results at both exposure times, and the cytotoxicity was between 30% and 55%, depending on the concentration. 

The IC_50_ values assessed in MTT assay were calculated from dose-response curves (Table 6). The most cytotoxic effects were induced by dust from grain dust collected from the blower elements transporting freshly harvested wheat from the field to the silo (No. 5): the IC_50_ was equal to 6.9 and 3.8 mg/mL after 48 and 72 h exposure of cells, respectively. Samples of dust originating from poultry farm (a livestock room with laying hens, No. 4) demonstrated the lowest cytotoxicity, and it was feasible to estimate IC_50_ only after 72-h exposure, which was 12.9 mg/mL. Approximate results were achieved for both cement plants (No. 1 and 2), where the IC_50_ values after 72-h exposure were 7.1 and 8.1 mg/mL. 

As exposure to dust and PM may occur through inhalation, toxicological research is essential to evaluate the risks derived from PM. Epidemiological studies indicate, that the high PM level (particularly respiratory PM < 10 µm) is associated with mortality or cardiovascular diseases [2]. The particles with diameter larger than 2.5 µm can be directly swallowed or finally reach the gastrointestinal tract after a short stay in tracheal and bronchial regions via inhalation. More than 80% of particles smaller than 2.5 µm can reach the pulmonary alveoli via inhalation, where they can be deposited and stay for months to years. Long-term exposure to PM can result in chronic pulmonary inflammation and increases the risk of developing pulmonary diseases, including asthma and chronic obstructive pulmonary diseases [2].

The present study demonstrated that exposure to grain dust induced the strongest cytotoxicity. This effect corresponds with the dust particle size fraction. In that sample either PM_1_ and PM_2.5_, or total PM content was the highest of all tested workplaces. This effect provides harmful, mass-dependent cytotoxic responses to small particle fractions.

Furthermore, the total number of bacteria, including actinomycetes and *P. fluorescens* bacteria, was also highest, and fungi, which can produce mycotoxins, were also at a high level. Akhtar et al. in MTT also demonstrated the mass-dependent cytotoxicity of PM particles of different origins to A-549 cells [2]. The cell death rate and release of cytokines in the bronchial epithelial cell line BEAS-2B in response to the PM_2.5_ treatments were higher than those with PM_10_ [49]. Additionally, Happo et al. observed a correlation between particle size and cytotoxicity. These authors also detected an association between cytotoxicity and microbe content [50]. Viegas et al. in WST-1 assay, showed 40% cytotoxicity of dust from poultry feed industry and near 25% cytotoxicity of dust originated from the poultry pavilion but after 18-h exposure of human monocytic THP-1 cells [4]. In the present study, the cytotoxicity of dust from chicken farms reached higher than 40% after 72-h exposition. 

The water-soluble fraction is the most liable and available fraction of dust and PM. The cytotoxic effects on A-549 cells were dependent on exposure time, exposure concentrations and sample dust origin. Our study confirms the relationship of either particle size fraction or content of microorganism in the sample with strength of cytotoxicity.

It is worth emphasizing that the described results relate to exemplary cement plant, composting plant, poultry farm, and cultivated area workplaces. The results obtained indicate that similar environments should be the subject of further research on microbiological and dust hazards for employees. Particularly valuable will be medical tests which can contribute to the understanding of the cause-effect relationship between the level of microbial and dust contamination and occupational diseases.

## 4. Conclusions

Among the studied workplaces, the highest airborne dust concentration exceeding the OEL was noted for the cultivated area, and for the remaining examined workplaces (poultry farm, composting plant, cement plant), the dust concentration was lower than the OEL limits. Factors, such as the high concentration of carbon, C:N ratio and neutral pH of the dust samples from cultivated areas, poultry farms, and composting plants can promote the growth of microorganisms. The number of microorganisms in the dust samples varied: 3.8 × 10^2^–1.6 × 10^8^ CFU/g (bacteria) and 1.5 × 10^2^–6.5 × 10^6^ CFU/g (fungi). The highest number of microorganisms was recorded in dust samples from cultivated areas (total number of bacteria, actinomycetes, and *P. fluorescens*) and composting plants (xerophilic fungi, and staphylococci); the least number of microorganisms was noted in the dust samples from cement plants (*p* < 0.05). Based on the metagenomics analysis, the biodiversity in the dust samples varied. Many types of potentially pathogenic microorganisms have been identified, including bacteria, such as *Bacillus*, *Actinomyces*, *Corynebacterium* (cement plants), *Prevotella*, *Clostridium* (poultry farms), and *Rickettsia* (cultivated area), and fungi, such as *Alternaria* (poultry farms and cultivation areas), *Cladosporium* (cement plants and poultry farms), *Penicillium* (cement plants and composting plants), and *Aspergillus* (all tested dust samples). The most cytotoxic (cytotoxicity 92.4%) to the human epithelial lung cell line A-549 was dust sample from the cultivated area (IC_50_ = 3.8 mg/mL after 72 h), and lower cytotoxicity was noted for the following dust samples obtained from: cement plants, composting plants and poultry farms. The cytotoxicity of the tested dust samples depends on the concentration of PM, the organic fraction with a high number of microorganisms, including numerous potentially pathogenic genera, and the exposure time.

## Figures and Tables

**Table 1:** Description of tested workplaces and collected dust samples.

Sample No. (Workplace)	Dust Sample	Workplace (Location/Coordinates)	Workplace Description (No. of Workers/Establishment Year/Machinery)	Measurement Date (Season)
1 (Cement plant)	Dust collected in the alternative fuel hall	Cement plant (Chełm, Lublin providence/51°21′01.2″ N 23°32′33.4″ E)	1–4/1960/waste homogenizer/tractor with bucket	11 July 2017 (early summer)
2 (Cement plant)	Dust collected in the clinker transporting conveyor hall		1–3/1960/clinker transporting conveyor	
3 (Composting plant)	Dust from a composting plant collected in the homogenisation hall of waste	Composting plant (Łódź, Łódź Province/51°43′39.5″ N 19°20′55.5″ E)	1–5/2014/waste homogenizer/ tractor with bucket	9 September 2017 (late spring)
4 (Poultry farm)	Dust collected in a livestock room—laying hens	Poultry farm (Zgierz, Łódź Province/51°51′15.4″ N 19°20′33.9″ E)	1–5/1980/manure transporting conveyor	7 April 2017 (early spring)
5 (Cultivated area)	Dust from grain collected from the blower elements transporting freshly harvested wheat from the field to the silo	Cultivated area (Budy, Łódź Province/51°51′15.4″ N 19°20′33.9″ E)	1–3/1995/tractor, agricultural trailer, combine-harvester, blower, silo	5 August 2017 (high summer)

**Table 2:** Microclimate conditions during measurements at the tested workplaces.

Sample No. (Workplace)	Temperature (°C)	Relative Humidity (%)	Air Flow Velocity (m/s)
1 (Cement plant)	M: 29.2	M: 53.0	M: 1.76
	SD: 0.1	SD: 0.2	SD: 0.26
	Min: 29.1	Min: 52.7	Min: 1.48
	Max: 29.4	Max: 53.3	Max: 2.05
2 (Cement plant)	M: 32.5	M: 43.8	M: 0.97
	SD: 0.2	SD: 0.7	SD: 0.07
	Min: 32.3	Min: 43.1	Min: 0.87
	Max: 32.8	Max: 44.8	Max: 1.05
3 (Composting plant)	M: 9.9	M: 51.1	M: 0.11
	SD: 0.6	SD: 3.5	SD: 0.08
	Min: 9.0	Min: 47.1	Min: 0.01
	Max: 10.7	Max: 56.6	Max: 0.21
4 (Poultry farm)	M: 18.1	M: 39.6	M: 0.50
	SD: 0.5	SD: 0.7	SD: 0.24
	Min: 17.5	Min: 38.9	Min: 0.09
	Max: 18.6	Max: 40.3	Max: 0.72
5 (Cultivated area)	M: 31.4	M: 29.6	M: 1.58
	SD: 1.0	SD: 2.2	SD: 0.09
	Min: 30.3	Min: 26.9	Min: 1.45
	Max: 32.9	Max: 31.9	Max: 1.70

M—mean, SD—Standard deviation, Min—minimum; Max—maximum.

**Table 3:** Airborne dust concentrations at selected workplaces.

Sample No. (Workplace)	Airborne Dust Concentrations Corresponding to Particle Size Fractions (mg/m^3^)				
	PM_1_	PM_2.5_	PM_4_	PM_10_	PM_total_
1 (Cement plant)	M: 0.246	M: 0.248	M: 0.251	M: 0.269	M: 0.282
	Med: 0.234 ^A^	Med: 0.236 ^A^	Med: 0.239 ^A^	Med: 0.256 ^A^	Med: 0.264 ^A^
	SD: 0.050	SD: 0.050	SD: 0.051	SD: 0.057	SD: 0.070
	Min: 0.220	Min: 0.221	Min: 0.222	Min: 0.229	Min: 0.231
	Max: 0.807	Max: 0.808	Max: 0.811	Max: 0.841	Max: 0.842
2 (Cement plant)	M: 0.639	M: 0.657	M: 0.755	M: 1.771	M: 2.076
	Med: 0.321 ^A^	Med: 0.328 ^A^	Med: 0.360 ^A^	Med: 0.626 ^A^	Med: 0.697 ^A^
	SD: 0.618	SD: 0.637	SD: 0.748	SD: 2.086	SD: 2.531
	Min: 0.232	Min: 0.234	Min: 0.238	Min: 0.287	Min: 0.309
	Max: 5.110	Max: 5.140	Max: 5.260	Max: 10.400	Max: 12.900
3 (Composting plant)	M: 0.269	M: 0.278	M: 0.290	M: 0.323	M: 0.334
	Med: 0.263 ^A^	Med: 0.271 ^A^	Med: 0.282 ^A^	Med: 0.310 ^A^	Med: 0.318 ^A^
	SD: 0.032	SD: 0.037	SD: 0.044	SD: 0.062	SD: 0.070
	Min: 0.229	Min: 0.233	Min: 0.236	Min: 0.243	Min: 0.243
	Max: 0.511	Max: 0.568	Max: 0.632	Max: 0.740	Max: 0.805
4 (Poultry farm)	M: 0.953	M: 0.981	M: 1.094	M: 1.888	M: 3.478
	Med: 0.897 ^A^	Med: 0.925 ^A^	Med: 1.040 ^A^	Med: 1.820 ^A^	Med: 3.305 ^A^
	SD: 0.257	SD: 0.257	SD: 0.261	SD: 0.356	SD: 0.873
	Min: 0.573	Min: 0.600	Min: 0.705	Min: 1.210	Min: 1.990
	Max: 2.610	Max: 2.630	Max: 2.730	Max: 3.690	Max: 8.200
5 (Cultivated area)	M: 27.572	M: 27.718	M: 28.401	M: 38.035	M: 46.019
	Med: 6.085 ^A^	Med: 6.440 ^A^	Med: 6.990 ^A^	Med: 14.100 ^A^	Med: 18.000 ^A^
	SD: 37.107	SD: 37.299	SD: 38.206	SD: 51.896	SD: 62.426
	Min: 1.170	Min: 1.210	Min: 1.250	Min: 1.640	Min: 1.640
	Max: 114.000	Max: 115.000	Max: 116.000	Max: 145.000	Max: 150.000

M—mean, Med—median, SD—Standard deviation, Min—minimum; Max—maximum, A: with the same capital letter in the same column are not significantly different (Dunn’s test; *p* < 0.05).

**Table 4:** Elemental composition and pH of settled dusts.

Sample No. (Workplace)	Composition (%)						pH
	C	N	H	P	S	C:N	
1 (Cement plant)	3.4	0.89	2.1	0.04	0.01	3.82	6.82
2 (Cement plant)	1.5	0.14	1.4	0.01	0.01	10.71	11.2
3 (Composting plant)	58.2	0.59	5.4	0.18	0.02	98.64	6.0
4 (Poultry farm)	71.2	4.9	5.9	2.54	0.93	14.53	5.95
5 (Cultivated area)	54.1	1.86	5.1	0.11	0.02	29.09	6.30

**Table 5:** Microorganism number in settled dust samples.

Sample No. (Workplace)	Microorganism Number [CFU/g]							
	Bacteria	Actinomycetes	Mannitol-Positive and Mannitol-Negative *Staphylococci* spp.	*Enterobacteriaceae*	*Pseudomonas fluorescens*	Haemolytic Staphylococcus	Fungi	Xerophilic Fungi
1 (Cement plant)	M: 2.88 × 10^7 A^	M: <1.00 × 10^2 A^	M: 2.01 × 10^6 A^	M: 3.81 × 10^4 B^	M: 2.75 × 10^3 A^	M: 2.88 × 10^6 A,B^	M: 4.48 × 10^6 A^	M: 2.30 × 10^5 A^
	SD: 1.13 × 10^7^	SD: 0	SD: 9.43 × 10^4^	SD: 2.37 × 10^4^	SD: 3.84 × 10^3^	SD: 2.09 × 10^6^	SD: 1.20 × 10^6^	SD: 2.07 × 10^5^
2 (Cement plant)	M: 3.75 × 10^2 A^	M: <1.00 × 10^2 A^	M: 1.00 × 10^2 A^	M: <1.00 × 10^2 A^	M: <1.00 × 10^2 A^	M: 1.00 × 10^3 A^	M: 1.50 × 10^2 A^	M: 1.50 × 10^2 A^
	SD: 1.89 × 10^2^	SD: 0	SD: 0	SD: 0	SD: 0	SD: 0	SD: 5.77 × 10^1^	SD: 1.00 × 10^2^
3 (Composting plant)	M: 2.80 × 10^7 A^	M: <1.00 × 10^2 A^	M: 1.07 × 10^7 B^	M: 1.07 × 10^4 A,C^	M: 2.50 × 10^4 A,B^	M: 4.85 × 10^6 B^	M: 6.48 × 10^6 A^	M: 3.45 × 10^6 B^
	SD: 8.02 × 10^6^	SD: 0	SD: 4.39 × 10^6^	SD: 4.94 × 10^3^	SD: 3.33 × 10^4^	SD: 3.13 × 10^6^	SD: 1.73 × 10^6^	SD: 2.36 × 10^6^
4 (Poultry farm)	M: 3.33 × 10^7 A^	M: <1.00 × 10^2 A^	M: 3.49 × 10^4 A^	M: <1.00 × 10^2 A^	M: 1.00 × 10^5 A^	M: 8.50 × 10^5 A^	M: 7.48 × 10^4 A^	M: 3.35 × 10^5 A^
	SD: 7.09 × 10^6^	SD: 0	SD: 2.78 × 10^4^	SD: 0	SD: 1.63 × 10^4^	SD: 2.08 × 10^5^	SD: 6.40 × 10^3^	SD: 2.54 × 10^4^
5 (Cultivated area)	M: 1.57 × 10^8 B^	M: 2.05 × 10^3 B^	M: 1.20 × 10^4 A^	M: 2.30 × 10^4 B,C^	M: 7.30 × 10^5 C^	M: 2.98 × 10^3 A^	M: 8.75 × 10^5 A^	M: 4.35 × 10^5 A^
	SD: 9.07 × 10^7^	SD: 1.55 × 10^3^	SD: 2.16 × 10^3^	SD: 4.76 × 10^3^	SD: 1.16 × 10^5^	SD: 1.34 × 10^3^	SD: 1.50 × 10^5^	SD: 2.45 × 10^5^

M—mean; SD—standard deviation; A, B, C—means with the same capital letter in the same column are not significantly different (Tukey’s test; *p* < 0.05).

**Table 6:** Cytotoxicity (IC_50_ values) of water-soluble fraction of settled dust samples of different origin estimated in MTT assay by using the human epithelial lung carcinoma cell line A-549.

Sample No. (Workplace)	IC_50_ [mg/mL]	
	48 h	72 h
1 (Cement plant)	9.0	7.1
2 (Cement plant)	not detected	8.1
3 (Composting plant)	9.3	10.5
4 (Poultry farm)	not detected	12.9
5 (Cultivated area)	6.9	3.8

## Supplementary Materials

The following are available online at http://www.mdpi.com/1660-4601/15/5/877/s1, Table S1: Archeal/bacterial diversity in dust samples, Table S2: Fungal diversity in dust samples.
