# Supplementary material for: Dust at Various Workplaces—Microbiological and Toxicological Threats
Source: Int J Environ Res Public Health. 2018 Apr 27;15(5):877. doi: 10.3390/ijerph15050877 (PMC5981916; doi:10.3390/ijerph15050877)
Supplement: Supplementary file 1 [file ijerph-15-00877-s001.pdf]

International Journal of
Environmental Research
and Public Health

MDPI

Supplementary Materials: Dust at Various Workplaces—Microbiological and Toxicological Threats

Beata Gutarowska [1], Justyna Szulc [1,*], Adriana Nowak [1], Anna Otlewska [1], Małgorzata Okrasa [2], Anita Jachowicz [1] and Katarzyna Majchrzycka [2]

Table S1. Archeal/bacterial diversity in dust samples.

Domain	Phylum	Genera	Abundance of archeal/bacterial genera in dust samples (%)*			
			1	3	4	5
**Archaea**	*Euryarchaeota*	*Methanobrevibacter*	0.000	0.000	0.052	0.000
		Methanosarcina	0.000	0.009	0.000	0.000
**Bacteria**	*Actinobacteria*	*Actinomyces*	7.370	0.701	0.252	0.023
		Arcanobacterium	0.064	0.000	0.000	0.000
		Actinokineospora	0.348	0.005	0.000	0.004
		Kibdelosporangium	0.021	0.000	0.000	0.000
		Salana	0.004	0.006	0.004	0.005
		Georgenia	0.144	0.070	0.000	0.000
		Brevibacterium	1.195	1.325	0.087	0.002
		Actinotalea	0.053	0.027	0.012	0.000
		Cellulomonas	0.006	0.060	0.000	0.000
		Demequina	0.010	0.037	0.000	0.000
		Oerskovia	0.027	0.031	0.001	0.000
		Corynebacterium	12.992	0.022	2.005	0.008
		Brachybacterium	3.397	1.433	0.133	0.001
		Dietzia	0.455	0.021	0.020	0.002
		Geodermatophilus	0.034	4.828	0.002	0.070
		Modestobacter	0.000	0.686	0.001	0.000
		Glycomyces	0.034	0.000	0.000	0.000
		Gordonia	0.135	0.006	0.000	0.000
		Kineococcus	0.008	0.039	0.003	0.174
		Curtobacterium	0.303	7.983	0.110	4.799
		Leucobacter	0.099	0.131	0.041	0.020
		Microbacterium	0.267	0.528	0.003	0.038
		Rathayibacter	0.002	0.005	0.003	0.203
		Salinibacterium	0.558	1.732	0.002	0.107
		Arthrobacter	0.098	9.281	0.019	0.002
		Kocuria	0.445	9.192	0.243	0.008
		Nesterenkonia	0.038	0.000	0.001	0.000
		Rothia	0.010	0.000	0.000	0.000
		Actinoplanes	0.01	0.03	0.00	0.00
		Mycobacterium	0.087	0.145	0.000	0.003
		Nocardia	0.029	0.254	0.000	0.000
		Rhodococcus	0.088	0.151	0.001	0.324
		Actinopolymorpha	0.085	1.524	0.004	0.030
		Aeromicrobium	0.009	1.066	0.002	0.075
		Friedmanniella	0.001	0.182	0.000	0.006
		Nocardioides	0.012	0.110	0.000	0.016
		Prauseria	0.432	0.002	0.000	0.000
		Thermobifida	0.466	0.036	0.000	0.000
		Luteococcus	0.008	0.030	0.001	0.000

	Microlunatus	0.001	0.016	0.000	0.000
	Tessaracoccus	0.010	0.008	0.001	0.001
	Actinomycetospora	0.082	0.078	0.003	0.001
	Amycolatopsis	0.039	0.016	0.000	0.000
	Prauserella	0.671	0.005	0.000	0.000
	Pseudonocardia	0.020	0.100	0.001	0.001
	Saccharomonospora	3.674	0.075	0.000	0.000
	Saccharopolyspora	0.635	0.131	0.001	0.004
	Thermobispora	0.086	0.135	0.000	0.000
	Thermocrispum	0.042	0.006	0.000	0.000
	Sanguibacter	0.147	0.929	0.009	0.160
	Streptomyces	0.179	0.096	0.001	0.000
	Nonomuraea	0.008	0.250	0.000	0.000
	Actinomadura	0.098	0.302	0.000	0.000
	Williamsia	0.011	0.019	0.001	0.040
	Yaniella	0.018	0.000	0.000	0.000
	Bifidobacterium	0.095	0.002	4.647	0.002
	Adlercreutzia	0.002	0.000	6.587	0.001
	Atopobium	0.000	0.000	0.018	0.000
	Collinsella	0.000	0.000	0.932	0.000
	Slackia	0.000	0.000	0.168	0.000
	Patulibacter	0.003	0.175	0.000	0.005
Bacteroidetes	*Bacteroides*	0.025	0.000	5.960	0.001
	Parabacteroides	0.102	0.000	0.762	0.000
	Prevotella	0.004	0.003	8.523	0.001
	Odoribacter	0.000	0.000	0.056	0.000
	Adhaeribacter	0.012	0.539	0.001	0.000
	Dyadobacter	0.010	0.096	0.001	0.015
	Hymenobacter	0.001	0.346	0.020	1.927
	Pontibacter	0.000	2.284	0.000	0.000
	Aequorivita	0.204	3.719	0.003	0.001
	Flavobacterium	0.028	2.253	0.002	0.012
	Gillisia	0.001	0.661	0.000	0.000
	Myroides	0.128	0.000	0.000	0.000
	Zhouia	0.027	0.000	0.000	0.000
	Chryseobacterium	0.088	0.102	0.066	0.065
	Cloacibacterium	0.020	0.000	0.000	0.000
	Wautersiella	0.054	0.002	0.012	0.000
	Olivibacter	0.452	6.841	0.041	0.038
	Parapedobacter	0.015	0.000	0.000	0.000
	Pedobacter	0.118	21.277	0.045	0.207
	Sphingobacterium	0.436	0.023	0.010	0.002
	Rubricoccus	0.001	0.033	0.000	0.000
	Flavisolibacter	0.000	0.271	0.000	0.001
	Segetibacter	0.000	0.522	0.000	0.000
Chloroflexi	*Thermomicrobium*	0.150	0.589	0.001	0.000
Cyanobacteria	*Phormidium*	0.130	0.105	0.759	19.704
Deferribacteres	*Mucispirillum*	0.000	0.000	0.159	0.000
Firmicutes	*Anoxybacillus*	0.023	0.000	0.000	0.000
	Bacillus	16.630	0.765	0.004	0.002
	Geobacillus	0.137	0.326	0.000	0.000
	Oceanobacillus	0.613	0.000	0.000	0.000
	Virgibacillus	0.403	0.001	0.001	0.000
	Brochothrix	0.029	0.002	0.000	0.000

Brevibacillus	0.134	0.049	0.000	0.000
Cohnella	0.001	0.010	0.000	0.000
Paenibacillus	0.073	0.023	0.009	0.474
Lysinibacillus	1.285	0.032	0.022	0.000
Planococcus	0.082	3.381	0.029	0.001
Solibacillus	0.006	0.005	0.014	0.001
Sporosarcina	0.064	0.008	0.007	0.005
Ureibacillus	0.843	0.057	0.000	0.000
Jeotgalicoccus	0.150	0.000	0.223	0.000
Macrococcus	0.043	0.000	0.003	0.000
Salinicoccus	0.035	0.000	0.000	0.000
Staphylococcus	3.022	0.038	0.333	0.007
Planifilum	0.563	0.399	0.000	0.001
Exiguobacterium	0.356	0.000	0.000	0.000
Aerococcus	14.985	0.023	0.914	0.001
Alkalibacterium	0.012	0.000	0.000	0.000
Facklamia	0.472	0.006	0.254	0.001
Marinilactibacillus	0.011	0.000	0.000	0.000
Carnobacterium	0.630	0.814	0.121	0.000
Desemzia	0.177	0.004	0.017	0.000
Trichococcus	0.030	0.022	0.146	0.000
Enterococcus	0.405	0.016	0.045	0.003
Lactobacillus	4.023	0.037	10.899	0.005
Pediococcus	0.302	0.007	0.000	0.000
Leuconostoc	1.674	0.071	0.145	0.003
Weissella	0.851	0.039	0.004	0.000
Lactococcus	0.169	0.004	0.001	0.000
Streptococcus	0.027	0.001	0.163	0.000
Turicibacter	0.002	0.001	0.248	0.000
Candidatus Arthromitus	0.000	0.000	0.019	0.000
Clostridium	0.117	0.056	8.571	0.001
Proteiniclasticum	0.018	0.000	0.097	0.000
Garciella	0.038	0.000	0.000	0.000
Eubacterium	0.000	0.000	0.030	0.000
Blautia	0.000	0.000	2.818	0.000
Clostridium	0.001	0.000	0.069	0.000
Coprococcus	0.019	0.003	0.310	0.000
Dorea	0.000	0.000	0.938	0.000
Ruminococcus	0.001	0.000	5.563	0.000
Peptococcus	0.000	0.000	0.290	0.000
Anaerofilum	0.028	0.000	4.835	0.001
Faecalibacterium	0.000	0.000	6.286	0.000
Oscillospira	0.000	0.000	1.082	0.000
Ruminococcus	0.017	0.000	0.331	0.000
Symbiobacterium	0.085	0.046	0.000	0.000
Dialister	0.000	0.000	0.817	0.000
Megamonas	0.000	0.000	4.607	0.000
Megasphaera	0.000	0.000	0.916	0.000
Phascolarctobacterium	0.000	0.000	0.568	0.000
Veillonella	0.017	0.000	0.016	0.000
Anaerococcus	0.091	0.001	0.071	0.000
Gallicola	0.000	0.000	0.069	0.000
Helcococcus	0.000	0.000	0.029	0.000
Peptoniphilus	0.000	0.000	0.072	0.000
Sporanaerobacter	0.023	0.000	0.000	0.000

	Tepidimicrobium	0.035	0.004	0.049	0.000
	Bulleidia	0.000	0.000	0.510	0.000
	Coprobacillus	0.000	0.000	0.314	0.000
	Eubacterium	0.000	0.000	0.724	0.000
Fusobacteria	*Fusobacterium*	0.002	0.000	4.355	0.001
Gemmatimonadetes	*Gemmatimonas*	0.045	0.113	0.000	0.000
	Brevundimonas	0.150	0.336	0.013	0.007
	Caulobacter	0.007	0.034	0.000	0.000
	Mycoplana	0.002	0.044	0.000	0.000
	Chelatococcus	0.074	0.016	0.000	0.000
	Balneimonas	0.001	0.002	0.000	0.000
	Ochrobactrum	0.120	0.006	0.004	0.006
	Devosia	0.086	1.993	0.001	0.005
	Rhodoplanes	0.000	0.000	0.000	0.000
	Methylobacterium	0.007	0.339	0.057	0.703
	Agrobacterium	0.027	0.064	0.007	0.192
	Paracoccus	0.262	0.178	0.011	0.000
	Rhodobacter	0.009	0.020	0.000	0.000
	Rubellimicrobium	0.006	0.008	0.000	0.000
	Inquilinus	0.030	0.000	0.000	0.000
	Skermanella	0.004	0.014	0.000	0.001
	Rickettsia	0.006	0.027	0.485	40.514
	Kaistobacter	0.110	1.044	0.003	0.063
	Novosphingobium	0.007	0.390	0.000	0.000
	Sphingobium	0.004	0.061	0.000	0.001
	Sphingomonas	0.040	0.921	0.069	4.948
	Pigmentiphaga	0.371	0.960	0.004	0.005
	Sutterella	0.000	0.000	0.387	0.000
	Acidovorax	0.023	0.657	0.002	0.026
	Comamonas	0.305	0.003	0.009	0.000
Proteobacteria	*Methylibium*	0.000	0.009	0.000	0.000
	Polaromonas	0.000	0.023	0.000	0.000
	Variovorax	0.001	0.080	0.003	0.069
	Janthinobacterium	0.107	0.745	0.061	1.154
	Ralstonia	0.080	0.006	0.008	0.008
	Methylotenera	0.000	0.100	0.000	0.000
	Desulfovibrio	0.000	0.000	0.242	0.000
	Myxococcus	0.012	0.000	0.000	0.000
	Campylobacter	0.000	0.000	0.021	0.000
	Helicobacter	0.000	0.000	0.064	0.000
	Zobellella	0.048	0.001	0.000	0.000
	Anaerobiospirillum	0.000	0.000	0.279	0.000
	Cellvibrio	0.031	0.216	0.001	0.000
	Marinobacter	0.305	0.000	0.000	0.000
	Idiomarina	0.046	0.087	0.000	0.000
	Pseudidiomarina	0.092	0.000	0.000	0.000
	Shewanella	0.123	0.001	0.000	0.000
	Rheinheimera	0.071	0.000	0.000	0.000
	Buchnera	0.000	0.000	0.000	0.012
	Erwinia	1.273	0.072	0.275	16.946
	Klebsiella	0.106	0.001	0.000	0.003
	Proteus	0.395	0.000	0.006	0.000
	Providencia	0.125	0.000	0.000	0.000
	Serratia	0.198	0.008	0.055	0.000
	Sodalis	0.000	0.000	0.000	0.298

		Alcanivorax	0.700	0.000	0.000	0.000
		Haererehalobacter	0.122	0.015	0.000	0.000
		Halomonas	0.420	0.002	0.000	0.000
		Avibacterium	0.000	0.000	0.014	0.000
		Gallibacterium	0.000	0.000	0.021	0.000
		Acinetobacter	3.675	0.292	0.996	0.010
		Alkanindiges	0.003	0.039	0.003	0.000
		Enhydrobacter	0.012	0.000	0.012	0.000
		Psychrobacter	0.226	0.206	0.004	0.001
		Pseudomonas	1.687	0.271	0.159	3.654
		Pseudoalteromonas	0.075	0.000	0.004	0.000
		Vibrio	0.029	0.000	0.000	0.000
		Luteibacter	0.208	0.004	0.010	0.134
		Lysobacter	0.103	0.176	0.000	0.000
		Pseudoxanthomonas	0.716	0.010	0.000	0.000
		Stenotrophomonas	0.217	0.000	0.002	0.012
	Verrucomicrobia	*Luteolibacter*	0.002	0.058	0.029	0.000
	Thermi	*Deinococcus*	0.000	0.092	0.065	0.000
		Thermus	0.012	0.000	0.000	0.000
**Other**	Other		0.097	0.085	4.842	1.596
**Unidentified** #	Unidentified		1.071	0.230	2.013	1.024

*percentage share of studied genera greater than 0.001%
#sequences that could not be classified into any phylogenetic group were assigned as unclassified.

Table S2. Fungal diversity in dust samples

Domain	Phylum	Genera	Abundance of fungal genera in dust samples (%)*				
			1	2	3	4	5
**Ascomycota**		*Dothiorella*	0.000	0.000	0.009	0.000	0.000
		Sphaeropsis	0.002	0.000	0.001	0.000	0.000
		Capnobotryella	0.000	0.000	0.274	0.000	0.000
		Rachicladosporium	0.000	0.000	0.016	0.000	0.000
		Cladosporium	2.351	0.000	14.134	18.718	0.613
		Cercospora	0.000	0.000	0.021	0.000	0.000
		Dissoconium	0.000	0.000	0.000	0.004	0.007
		Mycosphaerella	0.226	0.003	0.056	1.267	5.522
		Ramularia	0.008	0.000	26.820	0.038	0.000
		Devriesia	0.001	0.000	1.051	0.000	0.000
		Neodevriesia	0.000	0.000	0.007	0.004	0.000
		Teratosphaeria	0.000	0.000	1.809	0.001	0.001
		Aureobasidium	0.074	0.000	0.202	0.048	0.123
	Dothideomycetes	*Kabatiella*	0.025	0.000	0.004	0.000	0.000
		Monodictys	0.000	0.000	0.022	0.007	0.000
		Septoriella	0.001	0.000	0.000	0.003	0.017
		Zymoseptoria	0.011	0.000	0.007	0.008	0.043
		Pyrenochaetopsis	0.001	0.000	0.021	0.000	0.002
		Neoascochyta	0.001	0.000	0.064	0.035	0.718
		Angustimassarina	0.021	0.000	0.010	0.000	0.000
		Paraphaeosphaeria	0.000	0.000	0.003	0.001	0.006
		Phaeosphaeria	0.001	0.000	0.000	0.000	0.000
		Stagonospora	0.002	0.000	0.009	0.084	0.695
		Alternaria	0.301	0.000	0.547	1.466	7.336
		Bipolaris	0.000	0.000	0.000	0.006	0.003
		Drechslera	0.001	0.000	0.000	0.016	0.126
		Epicoccum	0.054	0.001	0.158	0.600	2.859

	Pyrenophora	0.016	0.000	0.001	0.135	0.400
	Stemphylium	0.004	0.000	0.033	0.028	0.132
	Ascochyta	0.000	0.000	0.025	0.000	0.000
	Boeremia	0.000	0.000	0.008	0.000	0.000
	Didymella	0.011	0.000	2.355	0.218	0.010
	Herpotrichia	0.000	0.000	0.010	0.000	0.000
	Setomelanomma	0.024	0.000	0.001	0.000	0.000
	Stagonosporopsis	0.000	0.000	0.017	0.000	0.000
Eurotiomycetes	*Arachnomyces*	0.022	0.000	0.000	0.000	0.000
	Cyphellophora	0.002	0.000	3.478	0.135	0.000
	Coniosporium	0.000	0.000	0.022	0.000	0.000
	Cladophialophora	0.000	0.000	0.000	0.000	0.000
	Exophiala	0.001	0.000	0.306	0.003	0.000
	Phaeococcomyces	0.010	0.000	0.011	0.054	0.026
	Thermomyces	9.234	0.005	1.442	0.002	0.003
	Thermoascus	0.326	0.000	0.123	0.000	0.000
	Aspergillus	34.884	0.012	10.227	1.449	0.035
	Byssochlamys	0.033	0.000	0.009	0.000	0.000
	Hamigera	0.015	0.000	0.000	0.000	0.000
	Paecilomyces	0.000	0.000	0.000	0.000	0.000
	Penicillium	6.623	0.000	0.236	0.090	0.002
	Phialosimplex	0.000	0.000	0.000	0.000	0.000
	Rasamsonia	0.155	0.000	0.088	0.000	0.000
	Sagenomella	0.030	0.000	0.000	0.000	0.000
	Talaromyces	4.045	0.002	0.105	0.054	0.001
	Monascus	0.083	0.000	0.008	0.029	0.000
	Chrysosporium	0.016	0.000	0.007	0.039	0.000
	Verrucaria	0.000	0.000	0.000	0.000	0.000
Lecanoromycetes	*Candelariella*	0.000	0.000	0.014	0.000	0.000
	Lepraria	0.000	0.000	0.006	0.000	0.002
	Peltigera	0.018	0.000	0.000	0.000	0.000
	Catillaria	0.010	0.000	0.000	0.000	0.000
	Umbilicaria	0.000	0.000	0.007	0.000	0.000
Leotiomycetes	*Blumeria*	0.020	0.000	0.005	0.001	0.001
	Erysiphe	0.004	0.365	0.000	0.000	0.000
	Crocicreas	0.000	0.000	0.012	0.000	0.000
	Scytalidium	0.000	0.000	0.000	0.002	0.000
	Cistella	0.000	0.000	0.007	0.000	0.000
	Proliferodiscus	0.010	0.000	0.000	0.000	0.000
	Phialocephala	0.000	0.000	3.058	0.000	0.000
	Malbranchea	0.062	0.000	0.015	0.000	0.000
	Pseudogymnoascus	0.234	0.000	0.766	0.015	0.000
	Lophodermium	0.112	5.006	0.010	0.030	0.000
	Thelebolus	0.000	0.000	0.009	0.000	0.000
Orbiliomycetes	*Arthrobotrys*	0.008	0.000	0.000	0.000	0.000
	Orbilia	0.280	0.000	0.001	0.000	0.000
Pezizomycetes	*Peziza*	0.031	0.000	0.000	0.000	0.000
	Terfezia	2.390	0.001	0.054	0.000	0.000
	Cephaliophora	1.226	0.000	0.000	0.001	0.002
	Cleistothelebolus	0.427	0.001	0.001	0.001	0.000
Pezizomycotina	*Chalara*	0.000	0.000	0.236	0.002	0.000
	Ciliophora	0.008	0.000	0.023	0.001	0.003
	Endophoma	0.000	0.000	0.047	0.002	0.001
	Gyoerffyella	0.001	0.000	0.088	0.000	0.000

	Knufia	0.000	0.000	0.246	0.000	0.000
	Megacapitula	0.000	0.977	0.000	0.000	0.000
	Ochroconis	0.011	0.000	0.000	0.000	0.000
	Olpitrichum	0.000	0.000	0.000	0.022	0.015
	Vestigium	0.020	0.000	0.000	0.000	0.000
Saccharomycetes	*Meyerozyma*	0.588	0.000	0.001	0.000	0.000
	Millerozyma	0.017	0.000	0.000	0.000	0.000
	Dipodascus	0.546	0.000	0.005	0.001	0.000
	Geotrichum	0.477	0.000	0.025	0.001	0.000
	Magnusiomyces	0.009	0.000	0.000	0.000	0.000
	Saprochaete	1.326	0.000	0.056	0.004	0.000
	Clavispora	0.362	0.000	0.001	0.000	0.000
	Metschnikowia	0.032	0.000	0.001	0.000	0.000
	Hyphopichia	0.264	0.000	0.001	0.002	0.000
	Pichia	1.035	0.000	1.028	0.008	0.000
	Kazachstania	0.041	0.000	0.000	0.000	0.000
	Kluyveromyces	0.054	0.000	0.000	0.000	0.000
	Saccharomyces	0.054	2.625	0.000	0.000	0.000
	Torulaspora	0.030	0.000	0.000	0.002	0.000
	Barnettozyma	0.070	0.000	0.000	0.000	0.000
	Candida	7.349	0.001	0.474	0.631	0.003
	Cyberlindnera	0.083	0.000	0.001	0.002	0.001
	Debaryomyces	1.191	0.000	0.001	0.004	0.001
	Kodamaea	0.125	0.000	0.000	0.000	0.000
	Wickerhamomyces	0.306	0.000	0.000	0.001	0.000
	Yarrowia	0.825	0.000	0.001	0.000	0.000
	Hanseniaspora	0.044	0.000	0.000	0.000	0.000
	Blastobotrys	0.885	0.000	0.000	0.002	0.000
	Trichomonascus	0.146	0.000	0.000	0.004	0.000
Sordariomycetes	*Coniochaeta*	0.028	0.000	0.000	0.000	0.000
	Pseudocatenomycopsis	0.000	0.106	0.001	0.001	0.000
	Beauveria	0.019	0.000	0.005	0.258	0.000
	Lecanicillium	0.063	0.000	0.001	0.003	0.032
	Acrostalagmus	0.004	0.000	0.004	0.000	0.000
	Sphaerostilbella	0.000	0.000	0.000	0.000	0.000
	Trichoderma	0.081	0.000	0.009	0.000	0.000
	Acremonium	0.562	0.001	3.321	2.655	0.027
	Calcarisporium	0.000	0.327	0.000	0.000	0.000
	Geosmithia	0.015	0.000	0.000	0.000	0.000
	Sarocladium	0.034	0.000	0.003	0.053	0.043
	Trichothecium	0.063	0.000	0.008	0.011	0.000
	Fusarium	0.113	0.000	0.028	0.437	0.924
	Neonectria	0.024	0.000	0.076	0.012	0.001
	Ophiocordyceps	0.000	0.000	0.000	0.000	0.000
	Gibellulopsis	0.000	0.000	0.294	0.000	0.000
	Harzia	0.000	0.000	0.001	0.009	0.000
	Sphaerodes	0.000	0.000	0.034	0.000	0.000
	Microascus	1.579	0.000	0.007	0.047	0.000
	Pseudallescheria	0.043	0.000	0.000	0.001	0.000
	Scedosporium	0.000	0.000	0.007	0.000	0.000
	Scopulariopsis	0.057	0.000	0.015	0.007	0.000
	Knoxdaviesia	0.000	0.000	0.000	0.000	0.000
	Ophiostoma	0.001	0.000	0.000	0.000	0.000
	Chaetomium	1.289	0.000	0.003	0.003	0.000
	Humicola	0.017	0.000	0.001	0.000	0.000

		Mycothermus	0.037	0.000	0.010	0.000	0.000
		Thielavia	0.223	0.000	0.112	0.038	0.000
		Sordaria	0.341	0.000	0.086	0.008	0.004
		Discosia	0.000	0.000	0.007	0.000	0.000
		Hypoxylon	0.011	0.000	0.008	0.000	0.000
		Monographella	0.022	0.000	0.033	0.021	0.121
	Taphrinomycetes	*Protomyces*	0.819	0.521	2.276	0.215	0.845
**Basidiomycota**	*Agaricomycetes*	*Coprinellus*	0.000	0.000	0.000	0.000	0.000
		Coprinopsis	0.285	0.000	0.002	0.000	0.000
		Hypholoma	0.031	1.356	0.001	0.001	0.001
		Amylocorticiellum	0.010	0.000	0.000	0.000	0.000
		Boletus	0.635	48.999	0.031	0.025	0.003
		Butyriboletus	0.000	0.000	0.000	0.000	0.000
		Rubroboletus	0.020	0.882	0.000	0.001	0.000
		Botryobasidium	0.012	0.000	0.000	0.000	0.000
		Sistotrema	0.035	1.086	0.021	0.004	0.000
		Corticium	0.000	0.000	0.000	0.000	0.000
		Vuilleminia	0.000	0.000	0.008	0.000	0.000
		Hyphodontia	0.022	0.627	0.001	0.000	0.000
		Steccherinum	0.000	0.692	0.000	0.000	0.000
		Phanerochaete	0.000	0.000	0.010	0.000	0.000
		Sistotremastrum	0.002	0.132	0.000	0.000	0.000
		Trechispora	0.000	0.000	0.010	0.000	0.000
	Agaricostilbomycetes	*Bensingtonia*	0.000	0.000	0.000	0.002	0.009
		Sterigmatomyces	0.001	0.000	0.237	0.383	0.000
		Kurtzmanomyces	0.000	0.000	0.264	0.000	0.000
		Kondoa	0.006	0.000	0.242	0.000	0.000
	Cystobasidiomycetes	*Cystobasidium*	0.316	0.000	0.072	0.006	0.000
		Buckleyzyma	0.001	0.000	0.022	0.001	0.001
		Symmetrospora	0.037	0.000	0.006	0.005	0.122
	Exobasidiomycetes	*Meira*	0.008	0.000	0.006	0.023	0.000
		Tilletia	0.000	0.000	0.001	0.159	0.028
	Microbotryomycetes	*Leucosporidiella*	0.000	0.000	0.007	0.000	0.001
		Leucosporidium	0.072	0.000	0.833	0.001	0.000
		Glaciozyma	0.011	0.000	0.000	0.000	0.000
		Sampaiozyma	0.051	0.000	0.000	0.000	0.000
		Rhodotorula	0.142	0.000	0.010	0.001	0.001
		Sporobolomyces	0.091	0.001	0.001	0.123	1.131
	Pucciniomycetes	*Septobasidium*	0.000	0.000	0.007	0.000	0.000
	Tremellomycetes	*Cystofilobasidium*	0.010	0.000	0.233	0.025	0.079
		Guehomyces	0.041	0.000	0.265	0.001	0.000
		Itersonilia	0.001	0.000	0.000	0.012	0.096
		Mrakia	0.047	0.000	0.013	0.004	0.000
		Udeniomyces	0.008	0.000	0.001	0.008	0.195
		Mrakiella	0.001	0.000	0.000	0.002	0.009
		Filobasidium	0.059	0.000	0.009	1.409	0.694
		Goffeauzyma	0.000	0.000	0.008	0.000	0.000
		Solicoccozyma	0.022	0.365	0.005	0.000	0.000
		Vishniacozyma	0.252	0.001	1.570	0.039	0.114
		Bullera	0.018	0.000	0.003	0.013	0.145
		Bulleromyces	0.000	0.000	0.003	0.003	0.012
		Cryptococcus	0.243	21.908	0.428	0.326	2.265
		Dioszegia	0.009	0.001	0.000	0.025	0.352
		Naganishia	0.077	0.000	0.180	0.037	0.000

		Tremella	0.021	0.000	0.079	0.003	0.031
		Apiotrichum	0.048	0.000	0.152	0.015	0.000
		Cutaneotrichosporon	0.226	0.000	0.081	0.650	0.000
		Trichosporon	0.224	0.000	0.106	4.859	0.000
		Vanrija	0.000	0.000	0.000	0.199	0.000
	Ustilaginomycotina	*Malassezia*	0.005	0.000	0.001	0.037	0.000
	Wallemiomycetes	*Wallemia*	0.108	0.000	0.006	1.089	0.000
**Glomeromycota**	*Glomeromycetes*	*Glomerales*	0.000	0.000	0.121	0.002	0.000
**Zygomycota**	*Kickxellomycotina*	*Spiromyces*	0.022	0.000	0.007	0.000	0.000
	Mortierellomycotina	*Mortierella*	0.242	0.000	0.024	0.001	0.000
	Mucoromycotina	*Lichtheimia*	0.090	0.000	0.000	0.000	0.000
		Rhizomucor	0.258	0.000	0.000	0.000	0.000
		Mucor	0.685	0.000	0.002	0.015	0.000
		Rhizopus	0.077	0.000	0.001	0.000	0.000
		Circinella	0.053	0.000	0.000	0.000	0.000
		Syncephalastrum	0.008	0.000	0.000	0.000	0.000
**Chromista**	*Ciliophora*	*Ciliophora*	0.119	0.605	0.477	0.200	0.045
**Plantae**	*Chlorophyta*	*Chlorophyta*	7.272	3.156	0.878	55.711	73.139
**Other**	Other		1.221	1.004	10.848	2.839	0.284
**Unidentified** #	Unidentified		1.957	9.230	6.399	2.692	0.531

*percentage share of studied genera greater than 0.001%

#sequences that could not be classified into any phylogenetic group were assigned as unclassified.

© 2017 by the authors. Submitted for possible open access publication under the terms and conditions of the Creative Commons Attribution (CC BY) license (http://creativecommons.org/licenses/by/4.0/).
